# Hardcore Heritage: Imagination for Preservation

**DOI:** 10.3389/fpsyg.2017.01995

**Published:** 2017-11-21

**Authors:** Erik Rietveld, Ronald Rietveld

**Affiliations:** ^1^Department of Philosophy, ILLC, University of Amsterdam, Amsterdam, Netherlands; ^2^Academic Medical Center, University of Amsterdam, Amsterdam, Netherlands; ^3^Amsterdam Brain & Cognition, University of Amsterdam, Amsterdam, Netherlands; ^4^Rietveld Architecture-Art-Affordances (RAAAF), Amsterdam, Netherlands

**Keywords:** cultural heritage, visual art, affordances (ecological psychology), built manifestos, strategic interventions, enactive architecture, radical embodied cognitive science, material art history

Should the practice of the historic preservation of built and landscape heritage necessarily be based on conservation? Monuments, listed buildings, landscapes, and other forms of built heritage, are typically regarded as immutable and untouchable—objects to be “conserved”—and as a result tend to fade from public imagination and memory (Rietveld et al., 2017). Current conservative preservation practices tend to fixate built heritage into an arbitrary historical state (choosing for example the building's early seventeenth century state as the reference), which negates both the historical process that shaped it (and followed it), as well as any possibility for rendering it relevant for the present or to bring us further into the future. Instead of just halting decay, we argue that one should aim at *generating meaning* from the old for current and future generations. In order to achieve this, we need a radically new perspective on built cultural heritage, which can only be reached by approaching heritage in a different way: one that conceives of preservation as *an effort toward imagination and activation*, rather than conservation.

This view has been the motivation behind RAAAF's radical interventions in the field of heritage. RAAAF [Rietveld Architecture-Art-Affordances] is a multidisciplinary studio operating at the intersection of architecture, visual art and philosophy. One example (Figure 1) of such a new perspective on cultural heritage is Bunker 599, where RAAAF|Atelier de Lyon cut through a bunker that is part of the UNESCO World Heritage-nominated New Dutch Waterline. In a radical way this intervention sheds new light on the Dutch and UNESCO policies on cultural heritage. At the same time, it makes people look at their surroundings in a new way. The bunker becomes the entrance to an 80 km long potential park of the twenty-first century, the New Dutch Waterline Park. A seemingly indestructible bunker with monumental status is sliced open. Paradoxically, after the intervention Bunker 599 became a Dutch national monument, so it “increased” in monumental value. The strategic intervention (Rietveld E. et al., 2014) offers a new perspective on the other 700 bunkers in the New Dutch Waterline.

**Figure 1:**
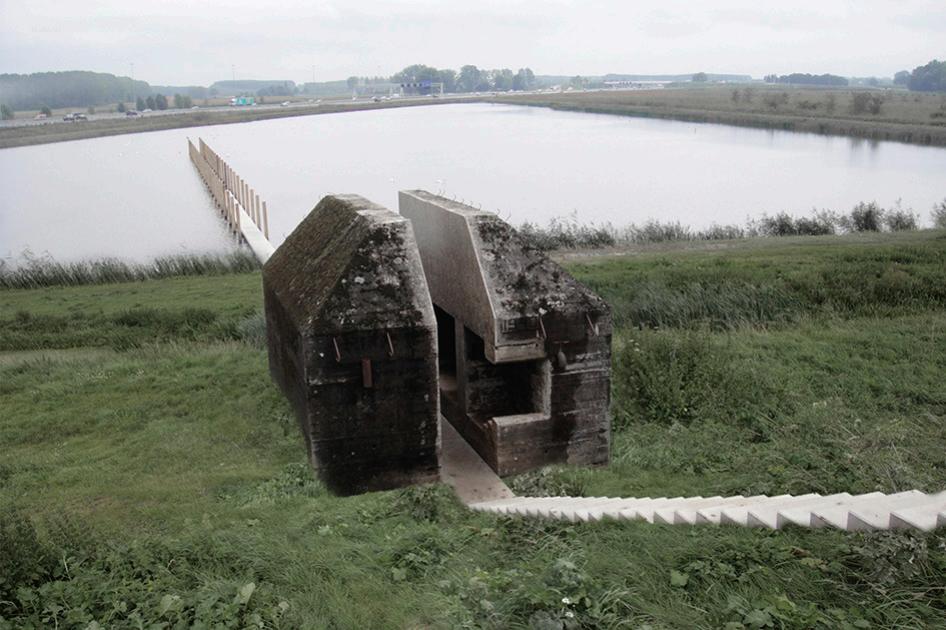
The sliced open Bunker 599 by RAAAF | Atelier de Lyon. Photo by: Allard Bovenberg.

Bunker 599 is a spatial thinking model; a vision on the future of the practice of historic preservation. Interventions like this avoid that we forget “a past that might trouble us” (Betsky, 2013). This dissected monument embodies and *imagination-based approach* toward preservation. The work:

– breaks through existing conventions;
– breaks through a seemingly indestructible structure;
– breaks though different disciplines; and
– breaks though past and present, looking for new meaning in the future.

We call this imagination-based approach “Hardcore Heritage.” Hardcore Heritage is a new way of thinking about monuments and cultural heritage, which started with RAAAF's built manifestos. Contrary to conservative historical preservation, this approach is not concerned with recreating or preserving the way an object might have looked liked in the past, e.g., in 1940 (which results in an ahistorical artifact), but rather focuses on generating meaning from multiple layers of history, meaning both for people now and in the future. Through deliberate destruction, radical changes in context, and seemingly contradictory additions, a new field of tension arises between present, past and future that activates built heritage, instead of “extracting” it from history and putting it on a pedestal. Such Hardcore Heritage interventions open up ways of *interpreting history toward the future*, rather than being stuck in fixated narratives from the past.

The spatial interventions of this approach vary per situation and context: it could be by removal, excavation, destruction, or alteration of buildings or sites. In any case, it results in new meaning and allows for a new appreciation of the special qualities and significance of the object. It affords people the possibility to discover material and immaterial qualities of their environment that would otherwise remain unnoticed.

Another example of how the Hardcore Heritage approach can—both literally and figuratively—unearth aspects of our environment that would otherwise stay hidden, can be found in RAAAF's project After Image. After Image allows people to experience an aspect of the Netherlands that is normally hidden from view: many Dutch cities are built on millions of pillars. After the radical demolition in 2011 of a sugar refinery that had been of great significance for the city of Groningen, almost nothing remained but a desolate field covered with grass. RAAAF was invited to make an artwork on this terrain nearby the city center of Groningen. Thanks to our research on the history of this location we discovered that the surface of the site—around which a new neighborhood will emerge in the coming years—covers a colossal city of pillars that lies hidden beneath it. Seven years after its demolition, the intervention reveals this underworld of the former sugar silo. By excavating the foundations of one of the former silos of the refinery, a concrete cathedral appears 30 feet (9 m) below ground level.

Hardcore Heritage flows from RAAAF's affordance-based approach (Rietveld and Rietveld, 2010; Rietveld and Kiverstein, 2014; Rietveld R. et al., 2014; Betsky, 2015; Rietveld et al., 2015). Affordances are possibilities for action provided by the environment (Gibson, 1979). More precisely, affordances are relations between aspects of the sociomaterial environment and abilities available in the human ecological niche (Rietveld and Kiverstein, 2014). Hardcore Heritage aims at providing affordances for spatial experiences that trigger one's imagination. By taking seriously the idea that people engage with their environment—such as heritage—based on the relevant affordances it offers to them, Hardcore Heritage provides a perspective than can clarify the value of cultural objects, by relating the use of objects in sociomaterial practices to the skills and concerns of people, instead of keeping objects at a distance the way conventional historic preservation tends to do.

In short, uncompromising confrontations between “conservation”, “destruction,” and “creation” lead to radically new ideas and spatial experiences. All means are permissible in Hardcore Heritage. Precisely because of deliberate imaginative degradation of built and landscape heritage, non-conservation like this can create a new field of tension between past, present, and future.

## Author contributions

ER initiated this piece. Both authors contributed equally to the text.

### Conflict of interest statement

The authors declare that the research was conducted in the absence of any commercial or financial relationships that could be construed as a potential conflict of interest.
